# Molecular genetic characterization analysis of a novel HIV-1 circulating recombinant form (CRF156_0755) in Guangdong, China

**DOI:** 10.3389/fmicb.2024.1387720

**Published:** 2024-05-03

**Authors:** Yaqing Lin, Xianglong Lan, Ruolei Xin, Xuemei Ling, Mingfeng Xiao, Feng Li, Fengyu Hu, Linghua Li, Yun Lan

**Affiliations:** ^1^Institute of Infectious Diseases, Guangzhou Eighth People's Hospital, Guangzhou Medical University, Guangzhou, China; ^2^Institute of AIDS/STD Prevention and Control, Beijing Center for Disease Prevention and Control, Beijing, China; ^3^Guangzhou Institute of Clinical Infectious Diseases, Infectious Disease Center, Guangzhou Eighth People’s Hospital, Guangzhou Medical University, Guangzhou, China; ^4^Guangdong Center for Diagnosis and Treatment of AIDS, Guangzhou, China

**Keywords:** HIV-1, CRF07_BC, CRF55_01B, NFLG, phylogenetic inference

## Abstract

**Introduction:**

The characteristic of human immunodeficiency virus type 1 (HIV-1) is its susceptibility to erroneous replication and recombination, which plays a crucial role in the diverse and dynamic variation of HIV-1. The spread of different subtypes in the same population often leads to the emergence of circulating recombination forms (CRFs). At present, the main recombinant subtypes of HIV-1 in China are CRF07_BC, CRF01_AE, CRF08_BC and B′ subtypes, while CRF55_01B has become the fifth major epidemic strain in China after rapid growth in recent years since it was first reported in 2013. In this study, we obtained five nearly full-length genomes (NFLGs) and one half-length genome from five different cities in Guangdong. Here, we focused on analyzing their characteristics, parental origin and drug resistance.

**Methods:**

Plasma samples were collected from six HIV-1 infected patients in Guangdong Province who had no epidemiological association with each other. The NFLGs of HIV-1 were amplified in two overlapping segments by the near-terminal dilution method. The positive products were sequenced directly to obtain genomic sequences. The recombinant patterns and breakpoints of the NFLGs were determined using the Simplot software and confirmed by the maximum likelihood trees for segments using the IQ-TREE and BEAST software. The genotypic resistance profiles of the protease reverse transcriptase and integrase were resolved by the Stanford HIV drug resistance database.

**Results:**

The six genomes shared highly similar recombinant pattern, with the CRF55_01B backbone substituted by CRF07_BC segments, therefore assigned as CRF156_0755. The evolutionary analysis of the segments showed that CRF07_BC segments were not clustered with the Chinese MSM variants in the CRF07_BC lineage. All the five NFLGs were identified with the non-nucleoside reverse-transcription inhibitors (NNRTIs) resistance mutation V179E.

**Discussion:**

With the accumulation and evolution of recombination between CRF55_01B and CRF 07_BC, the prevalence of more recombinant strains of CRF55_01B and CRF 07_BC may occur. Therefore, it is necessary to strengthen the identification and monitoring of the recombination of CRF55_01B and CRF 07_BC.

## Introduction

It has been 39 years since HIV was first reported in China in 1985. The national epidemic situation of legal infectious diseases in 2021 showed that 22,198 cases of Class A, B and C infectious diseases had died that year, of which 88.4% died of Acquired Immune Deficiency Syndrome (AIDS).^1^ Gene recombination is one of the important sources of high genetic diversity in the HIV genome. So far, 143 CRFs of HIV-1 have been identified.^2^ A systematic review indicated that CRF02_AG (33.90%), CRF01_AE (23.00%) and unique recombinant forms (URFs) (26.70%) were popular globally (Hemelaar et al., 2020). And the main subtypes of HIV-1 in China were CRF07_BC, CRF01_AE, CRF08_BC and B′ (Liu et al., 2023). A study (Lan et al., 2021) showed that the main subtypes of HIV-1 in Guangdong Province were CRF07_BC (35.90%), CRF01_AE (35.56%) and CRF55_01B (10.30%) in 2018. It can be seen that CRFs and URFs of HIV-1 are gradually becoming the main epidemic strains worldwide. The production of recombinant viruses may lead to an increase in virus virulence or infectivity, thereby accelerating the process of HIV-1 infection (Hemelaar et al., 2020).

Due to the fact that recombination occurs during the simultaneous infection of two or more viruses into the same cell, the emergence of a new recombinant virus often implies an event of mutual infection between populations infected with different subtypes (Pérez-Losada et al., 2015; Rawson et al., 2018). Breakpoint analysis of the recombinant virus and determining the source of the parental strain can help to understand the transmission events between different populations and thus gain a deeper understanding of the laws of disease transmission (Pérez-Losada et al., 2015).

In order to understand the characteristics of HIV-1 recombination in Guangdong Province and provide clues and references for the prevention and control of AIDS, this paper analyzed the characteristics of the NFLGs of six HIV-1 infected patients in Guangdong Province, and determined the breakpoint structure model and parental origin of the recombinant virus.

## Materials and methods

### Study population

In the previous pretreatment drug resistance surveillance among HIV-1 infected individuals without highly active antiretroviral therapy (HAART) in Guangdong province from 2018 to 2022, we found six out of all 10,340 partial pol gene sequences formed a distinct cluster separate from previously reported CRFs. Plasma specimens collected from these six HIV-1 infected patients (whose information was presented in Table 1), were used to identify a possible newly emerging CRF in this study. This study was approved by the institutional review board of the Guangzhou Eighth People’s Hospital (No. 202213225), all individuals provided written informed consent.

**Table 1:** Sociodemographic characteristics of six CRF156_0755 infected individuals.

Sequence name	Confirming time	Route	Sampling data	Patient sex	Sample city	Sequence length	HXB2 coordinate range	Accession number	Baseline CD4 (cells/μL)	Baseline VL (IU/mL)
ZLQ00277	2018/11/14	HET	2018/11/21	Male	Zhongshan	8,981 bp	644–9,375	OR570897	403	5.37E+06
ZLQ00974	2018/11/20	MSM	2018/11/27	Male	Foshan	8,981 bp	644–9,375	OR570896	286	6.12E+05
ZLQ02146	2019/5/16	HET	2019/5/21	Male	Zhaoqing	8,996 bp	644–9,375	OR581726	417	6.91E+06
190837_3’	2019/10/1	MSM	2019/10/25	Male	Guangzhou	4,694 bp	4,912–9,375	OR570895	308	6.89E+04
ZLQ05825	2021/4/13	MSM	2021/5/20	Male	Jiangmen	8,263 bp	644–8,864	OR581727	406	1.65E+04
220,002	2021/12/24	HET	2022/1/4	Male	Guangzhou	9,004 bp	644–9,375	OR581725	142	2.21E+05

HET, Heterosexual Transmission; MSM, Men who have sex with men.

### Nucleic acid extraction, amplification, sequencing and sequence assembly

Viral RNAs were extracted from plasma samples using an automatic magnetic bead-based Virus RNA Extraction Kit (Daan, China) and a semi-automatic nucleic acid extraction instrument Smart 32 (Daan, China), and subsequently reverse transcribed to cDNA using SuperScript III First-Strand Synthesis System (Invitrogen, United States). Then, two overlapping halves of HIV-1 genome were amplified by nested polymerase chain reaction (PCR) utilizing LA Taq (TaKaRa, China) with a near endpoint dilution method as described previously (Yao et al., 2021). Positive products were directly purified and sequenced by a commercial company (Tianyihuiyuan, Guangzhou). The NFLGs were assembled and cleaned by DNA sequence analysis software Sequencher V5.1 (Gene Codes, United States).

### Phylogenetic analysis of sequences

Five NFLGs and one half-length genome were obtained and deposited in GenBank to get accession numbers. Then aligned with reference sequences downloaded from the Los Alamos HIV database^3^ by GeneCutter tool,^4^ including 10 subtypes (A-D, F-H, and J-L), CRF01_AE, CRF07_BC, and CRF55_01B and performed by BioEdit V7.0. The Maximum likelihood (ML) phylogenetic tree was constructed using the general time reversible (GTR) substitution model with the ultrafast bootstrap value 1,000 to assess the reliability of internal branches in IQtree software V1.6.12.

### Analyze restructuring breakpoints

Using the Simplot function in SimPlot V3.5 software to perform Similarity Plot on the previously obtained alignment sequences. Based on the differences in similarity, selected parental reference sequences (CRF55_01B and CRF07_BC sequences) and background reference sequence (a N group sequence) for BootScan analysis. Using the FindSites function of the software to determine recombination breakpoints. The genome structure map of the recombinant virus was drawn using the Map Draw tool of the Los Alamos HIV sequence database according to the corresponding positions of the international standard strain HXB2. The schematic structure is created using the Recombinant HIV-1 Drawing Tool.^5^ The accuracy of the determined recombination breakpoints was further confirmed by constructing ML evolutionary trees using segmented methods, with appropriate addition of parental subtype sequences as a reference during analysis.

### Analyze the source of parental strains

In order to determine the possible sources of different subtype parent strains in the CRF156_0755, the Bayesian coalescent Markov Chain Monte Carlo (MCMC) analyses approach with a chain length of 200 million based on the concatenated segments were performed using BEAST software V1.10.4. A Gamma substitution model, an uncorrelated relaxed log-normal molecular clock model and a Bayesian skyline model were selected as the best-fit model for this analysis. The first 10% of states of each run were discarded as burn-in. The convergence of parameters was checked using Tracer v1.5. The Maximum Cladecredibility (MCC) tree was constructed by TreeAnnotator v1.7.2 after burning-in the first 10% of states of each run, and visualized by FigTree v1.3.1. The effective sample size (ESS) was >200.

### Drug resistance analyses

The HIVdb program from the Stanford University HIV Drug Resistance Database^6^ was used to analyze the sequences for drug resistance mutations (DRMs). DRMs were classified into the protease inhibitors (PIs), nucleoside reverse-transcription inhibitors (NRTIs), non-nucleoside reverse-transcription inhibitors (NNRTIs) and integrase strand transfer inhibitors (INSTIs). Drug resistance was divided into five levels (sensitive, potential low, low level, intermediate level, high level drug resistance), and sequences with low or greater resistance were defined as having drug resistance.

## Results

### Phylogenetic analysis of the NFLGs sequences

**Figure 1:**
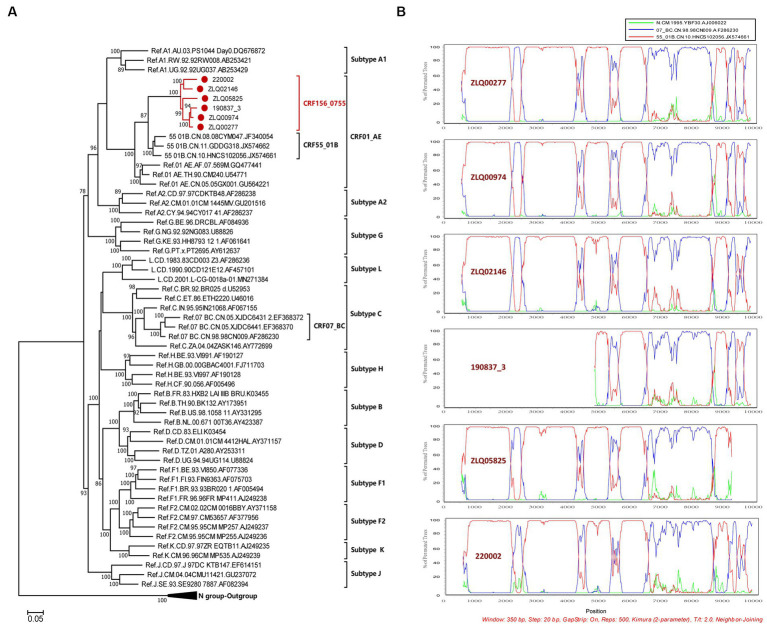
Phylogenetic and recombination analyses of CRF156_0755. **(A)** The ML phylogenetic tree was constructed using general time reversible (GTR) substitution model with the ultrafast bootstrap value 1,000 to assess the reliability of internal branches in IQtree software V1.6.12. The reference sequences were downloaded from the Los Alamos National Library (LANL) HIV database (https://www.hiv.lanl.gov/), including subtypes A-D, F-H, J, K, L, CRF01_AE, CRF07_BC, and CRF55_01B together with CRF156_0755 sequences. The sequences of CRF156_0755 were marked with red dot. More than 70% of the bootstrap values were categorized as reliable clusters and labeled at the nodes. **(B)** Bootscan analysis was conducted using a window size of 350 bp and a step size of 20 bp along with reference strains of CRF07_BC, CRF55_01B, and a representative HIV-1 N group sequence.

The NFLG sequences isolated from four individuals were 8,981, 8,981, 8,996, and 9,004 nt in size for strain ZLQ00277, ZLQ00974, ZLQ02146, and 220,002 respectively, spanning from 5′ long terminal repeats (LTR) to 3′ LTR corresponding to the location 644–9,375 nt of HXB2 strain. In addition, the 190837_3’ strain was 4,694 bp long and the ZLQ05825 strain was 8,263 bp long, spanning from the 5′ LTR to the 3′ LTR corresponding to locations 4,912–9,375 nt and 644–8,864 nt of the HXB2 strain, respectively. The ML phylogenetic tree revealed that these six sequences formed a tight, large, monophyletic cluster (bootstrap value of 100%) and were distinguished from known subtypes and CRFs, as shown in Figure 1A.

### Recombinant breakpoint analysis

**Figure 2:**
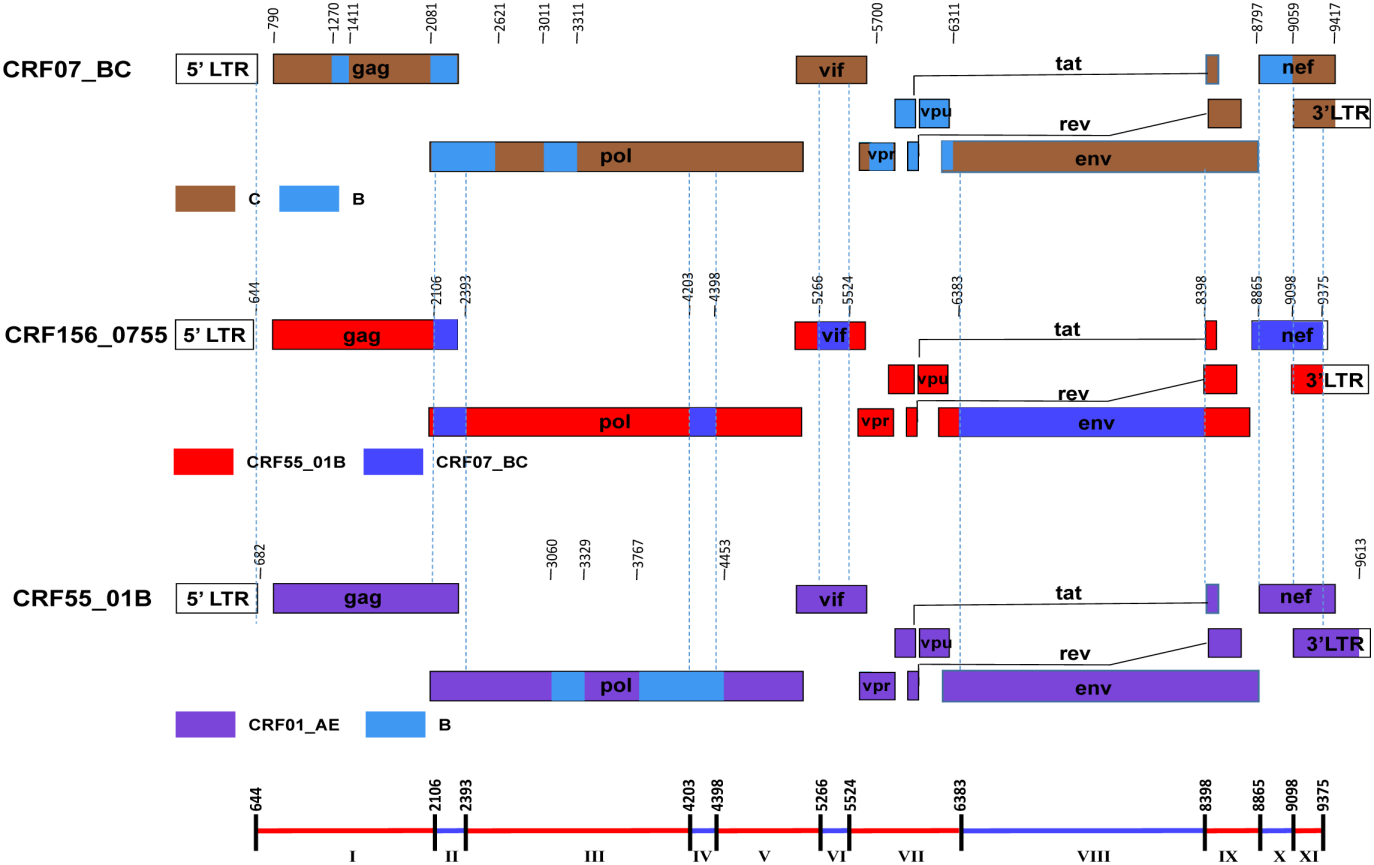
The genomic maps of CRF156_0755, comparied with its parental strain CRF07_BC and CRF55_01B. The genome mosaic map of CRF156_0755 was correspondingly drawn according to the LANL Recombinant HIV-1 Drawing Tool (https://www.hiv.lanl.gov/content/sequence/DRAW_CRF/recom_mapper.html).

The BootScan analysis revealed that the six sequences shared a highly similar recombinant pattern, with the corresponding segments in the CRF55_01B backbone substituted by subtype CRF07_BC (Figure 1B). The whole genome was divided into 11 regions with 10 breakpoints. The schematic representation of mosaic genomic structure of CRF156_0755 was generated using the Recombinant HIV-1 Drawing Tool from Los Alamos HIV Sequence Database (Figure 2). A total of 10 recombinant breakpoints were found at HXB2 positions according to the recombination analysis as follows: I (644–2,105 nt), II (2106–2,392 nt), III (2393–4,202 nt), IV (4203–4,397 nt), V (4398–5,265 nt), VI (5266–5,523 nt), VII (5524–6,382 nt), VII (6383–8,397 nt), IX (8398–8,864 nt), X (8865–9,097 nt), XI (9098–9,375 nt), using HXB2 as a reference (Figure 2).

### Subregion phylogenetic tree analysis

**Figure 3:**
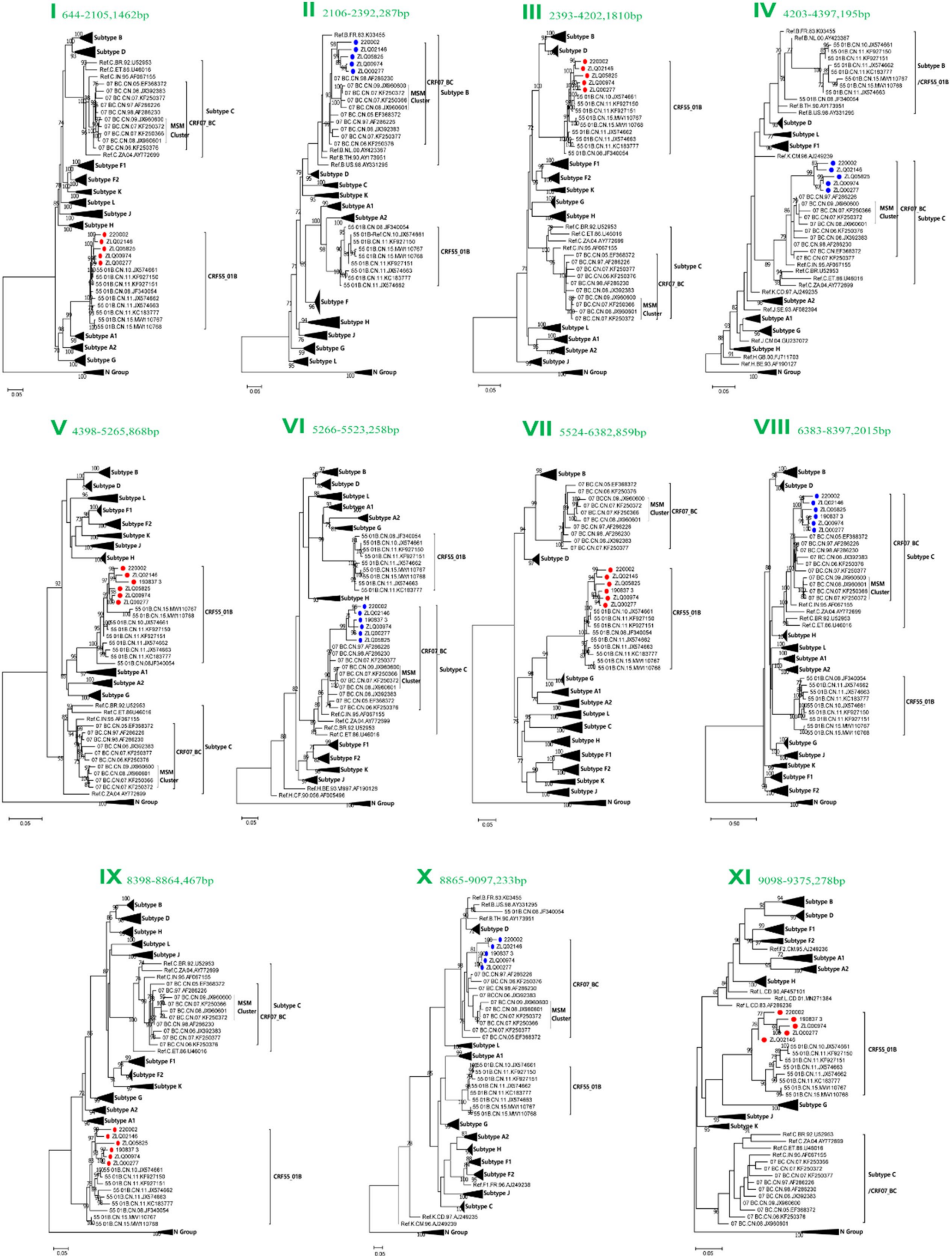
Subregion Maximum likelihood (ML) phylogenetic analysis and maximum clade credibility (MCC) trees of CRF156_0755. All the subregion ML trees were reconstructed using the same references, including a group N sequence, twelve subtypes (A1, A2, B-D, F1, F2, G-H, and J-L) and more Chinese CRF55_01B plus more Chinese CRF07_BC. The subregion ML trees were constructed using general time reversible (GTR) substitution model with the ultrafast bootstrap value 1,000 to assess the reliability of internal branches in IQtree software V1.6.12. More than 70% of the bootstrap values were categorized as reliable clusters and labeled at the nodes. Subregion phylogenetic analyses indicated that the segment I, III, V, VII, IX and XI of CRF156_0755 belonged to the CRF55_01B. The segment II, IV, VI, VII, and X of CRF156_0755 were clustered with the CRF07_BC cluster.

Subregion phylogenetic analyses of 11 genomic segments were further conducted to explore their likely parental lineages. The high bootstrap values in the phylogenetic tree supported a close relationship with CRF55_01B or CRF07_BC subtype references, respectively, (Figure 3). Subregion phylogenetic analyses indicated that the segment I, III, V, VII, IX and XI of CRF156_0755 belonged to the CRF55_01B. The segment II, IV, VI, VII, and X of CRF156_0755 were clustered with the CRF07_BC cluster, which is not originated from CRF07_BC MSM lineage.

**Figure 4:**
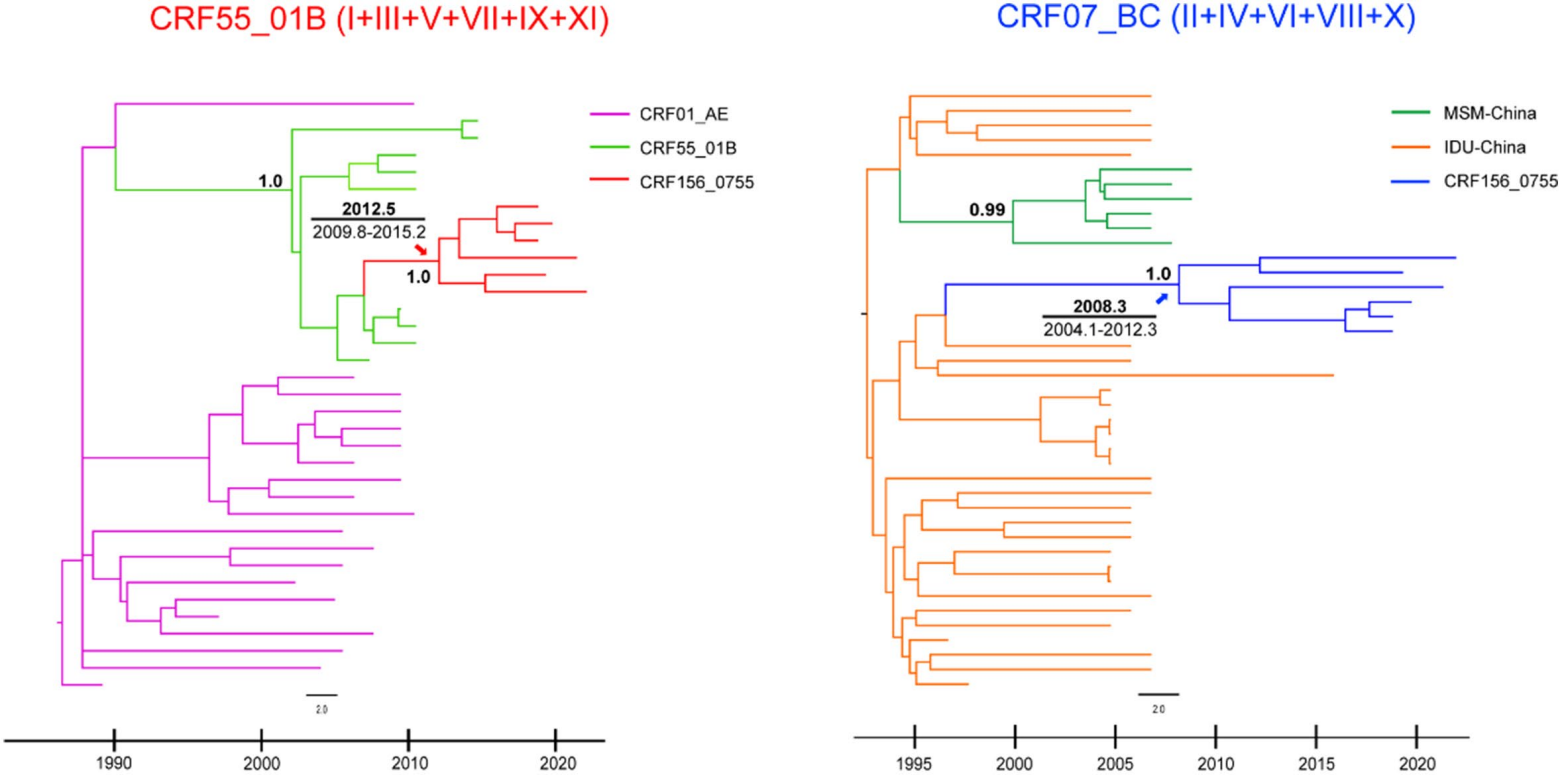
Bayesian phylogenetic analyses were performed using BEAST software V1.10.4. The MCC trees for CRF07_BC and CRF55_01B regions of CRF156_0755 were visualized and edited in FigTree 1.4.0. The mean tMRCA and 95% HPD for the key nodes were displayed in MCC trees.

The Bayesian phylogenetic analyses based on the concatenated segments I, III, V, VII, IX and XI pertaining to CRF55_01B and the segment II, IV, VI, VII, and X pertaining to CRF07_BC indicated that the estimated time of the most recent common ancestor (tMRCA) for CRF55_01B segment and CRF07_BC segment were 2012.4606 [95% highest probability density (HPD):2009.8439, 2015.1741] and 2008.2866 (95% HPD, 2004.0576, 2012.3348), respectively (Figure 4). Therefore, HIV-1 CRF156_0755 was inferred to approximately originated around 2008–2012. The evolutionary analysis of the segments also showed that CRF07_BC segments were not clustered with the MSM variants in the CRF07_BC lineage. Therefore, it was not derived from the MSM variants, but it may be derived from the IDU variants.

### Drug resistance mutations and resistance to ART drugs

Genotypic drug resistance was interpreted by the Stanford HIV Drug Resistance Database. No PI, NRTI or INSTI DRMs were identified. Five other NFLGs of the CRF156_0755 except 190837_3′ were identified with the NNRTI resistance mutation V179E, in which the codon in HXB2 was mutated from GTT to GAA. This mutation caused potential low-level resistance to efavirenz (EFV), etravirine (ETR), nevirapine (NVP) and rilpivirine (RPV).

## Discussion

Genetic diversity and genetic mutations are important characteristics that lead to the widespread spread and prevalence of HIV-1, and the continuous genetic recombination between different subtype strains is an important source of HIV-1 evolution (Lan et al., 2020). The distribution of HIV-1 genotypes has obvious population and regional characteristics. Therefore, the identification of new recombinant viruses and the analysis of the source of parental strains are of great significance for understanding the relationship between different transmission routes and different subtypes and discovering the characteristics of possible bridge populations.

It is widely believed that CRF07_BC originated from injecting drug users (IDUs) (Feng et al., 2016) in Yunnan and later spread to other regions of China, especially Beijing, Shanghai, Guangdong and Zhejiang (Vrancken et al., 2020). During the process of transmission epidemic, CRF07_BC was divided into two clusters: 07BC_O and 07BC_N. The 07BC_O is the original CRF07_BC, circulating in people who inject drugs (PWID) and heterosexuals, predominantly in southwestern and northwestern provinces of China. The 07BC_N is a new cluster, identified mostly in men having sex with men (MSM) in the northern provinces of China (Ge et al., 2021). However, the evolutionary analysis of the segments showed that CRF07_BC segments in CRF156_0755 were not clustered with the Chinese MSM variants in the CRF07_BC lineage. CRF 55_01B was first reported in 2013 among MSM from Changsha city of Hunan province and Dongguan city of Guangdong province in China and it was composed of CRF01_AE and subtype B. However, the earliest known strain of CRF55_01B was traced back to 2007 in sample from Shenzhen of Guangdong province among MSM. Now, CRF55_01B is mainly distributed in Guangdong and neighboring provinces in China, and is found across all risk groups (Zai et al., 2020).

**Figure 5:**
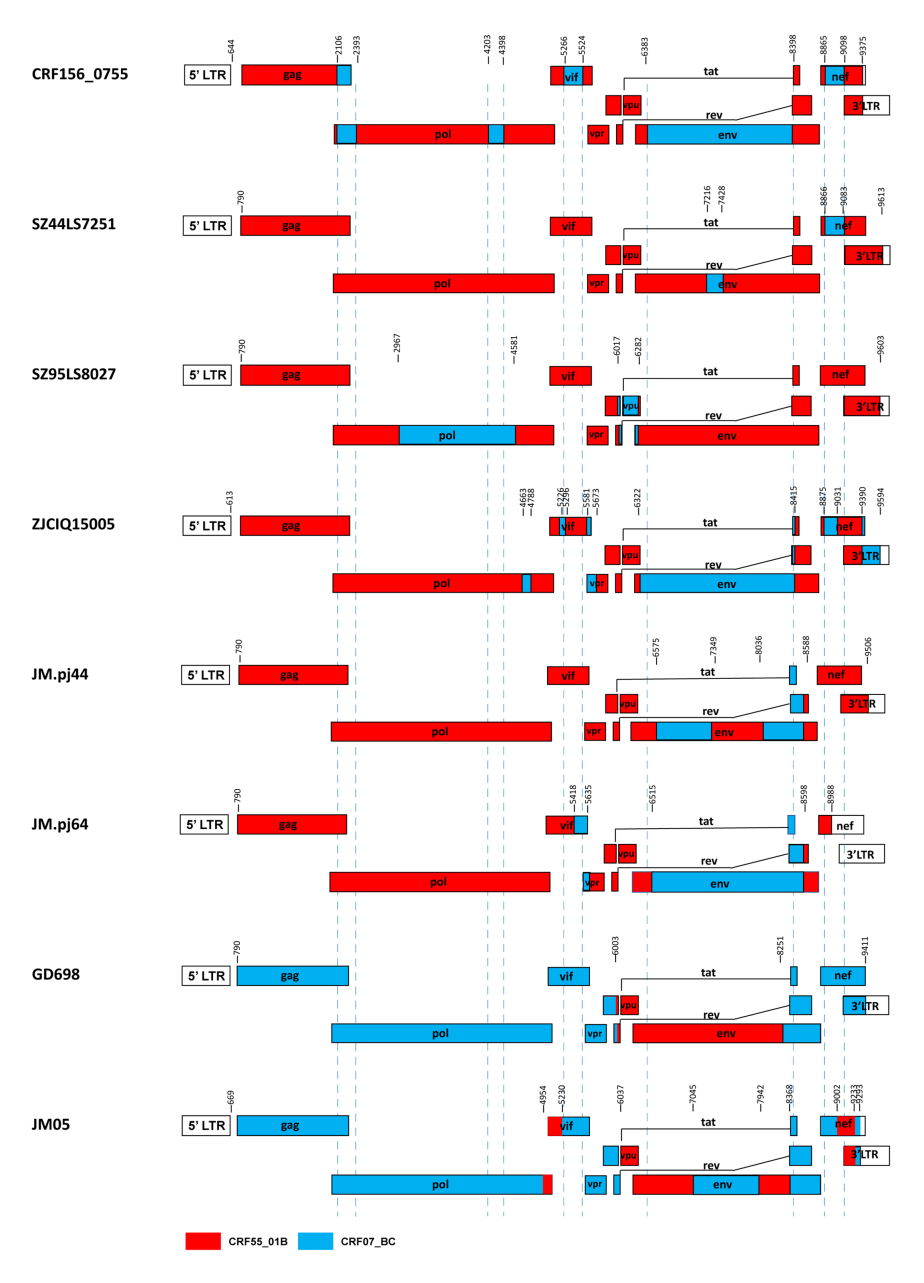
Comparison between CRF156_0755 and other recombinants of CRF07_BC/ CRF55_01B. The mosaic structures were created using the Recombinant HIV-1 Drawing Tool (https://www.hiv.lanl.gov/content/sequence/DRAW_CRF/recom_mapper.html).

Our results and the previous studies indicate that the recombination forms between the CRF07_BC and CRF55_01B are emerging. For example, Jia et al. reported two novel recombinants in which two small fragments of CRF07_BC inserted into the backbone sequence of CRF55_01B from heterosexual persons SZ44LS7251 and SZ95LS8027 in Shenzhen, China (Jia et al., 2017). Wang et al. reported a novel HIV-1 recombinant composed of CRF55_01B as the backbone and CRF07_BC with 12 recombinant break points observed in the pol, vif, vpr, tat, rev, env, nef, and 3’ LTR regions from a Malaysian immigrant worker ZJCIQ15005 in Zhejiang, China (Wang et al., 2017). Li et al. (2022) identified two novel HIV-1 CRF55_01B/CRF07_BC second-generation recombinant forms(SGRs)of JM. pj44 and JM. pj64, which were derived from homosexual and heterosexual groups in Jiangmen, China (Li et al., 2022). These URFs described above, like CRF156_0755, all kept the CRF55_01B parental backbone with CRF07_BC segments inserted. Furthermore, Guangzhou (Wu et al., 2017) and Jiangmen (Han et al., 2020) also reported two cases of recombinant strains with CRF55_01B segments inserted into the CRF07_BC backbone. The difference between CRF156_0755 and other CRF07_BC/CRF55_01B containing recombinants was observed based on the different mosaic structures of their genomes, as shown in Figure 5.

A study in 2019 has demonstrated that the natural presence of V179E mutations associated with resistance to non-nucleoside reverse transcriptase inhibitors was found in all but one of the 228 patients infected with CRF55_01B (Liu et al., 2020), which is consistent with our results. Guan et al. (2020) found that 17 cases with gene subtype CRF 55_01B developed mutation sites of V179E and E138G associated with drug resistance in Jiangmen, respectively. A study (Lan et al., 2020) on genotype drug resistance of 162 cases with CRF 55_01B patients who had received ART from 2013 to 2018 found that 79.01% of patients developed low to high level resistance to current antiviral drugs, among which V179E mutation accounted for 98.8%. The above studies showed that the level of resistance-related mutations occurring in CRF 55_01B strain is high, whether the V179E mutation is a polymorphic mutation or a selective mutation has not been determined.

In conclusion, we discovered a novel HIV-1 CRF in Guangdong Province, defined as CRF156_0755, with the CRF55_01B backbone substituted by CRF07_BC segments. Moreover, Bayesian coalescent inference indicated that CRF156_0755 emerged in approximately 2008–2012. The emergence of CRF156_0755 indicates that the recombination between CRF 07_BC and CRF 55_01B is constantly accumulating and evolving, more complex recombination patterns of CRF 07_BC and CRF 55_01B may appear over time, and it can be a challenge to develop drug resistance. Therefore, it is necessary to prepare for the situation preventing CRF 07_BC and CRF 55_01B coinfection, and reinforce identification and monitoring of CRF 07_BC recombination with CRF 55_01B.

## Data availability statement

The datasets presented in this study can be found in online repositories. The names of the repository/repositories and accession number(s) can be found at: https://www.ncbi.nlm.nih.gov/genbank/, OR570897 https://www.ncbi.nlm.nih.gov/genbank/, OR570896 https://www.ncbi.nlm.nih.gov/genbank/, OR581726 https://www.ncbi.nlm.nih.gov/genbank/, OR570895 https://www.ncbi.nlm.nih.gov/genbank/, OR581727 https://www.ncbi.nlm.nih.gov/genbank/, OR581725.

## Ethics statement

The studies involving humans were approved by Medical ethics committee, Guangzhou Eighth People’s Hospital, Guangzhou Medical University. The studies were conducted in accordance with the local legislation and institutional requirements. The participants provided their written informed consent to participate in this study.

## Author contributions

YaL: Data curation, Formal analysis, Writing – original draft, Writing – review & editing. XL: Formal analysis, Methodology, Writing – review & editing. RX: Data curation, Investigation, Writing – review & editing. XL: Investigation, Resources, Writing – review & editing. MX: Investigation, Resources, Writing – review & editing. FL: Investigation, Resources, Writing – review & editing. FH: Funding acquisition, Investigation, Project administration, Resources, Writing – review & editing. LL: Funding acquisition, Investigation, Project administration, Resources, Writing – review & editing. YuL: Funding acquisition, Investigation, Methodology, Project administration, Resources, Supervision, Writing – review & editing.

## Footnotes

^1^Available at: http://www.nhc.gov.cn/jkj/s3578/202204/4fd88a291d914abf8f7a91f6333567e1.shtml

^2^Available at: https://www.hiv.lanl.gov/components/sequence/HIV/search/search.html

^3^ https://www.hiv.lanl.gov

^4^ https://www.hiv.lanl.gov/content/sequence/GENE_CUTTER/cutter.html

^5^ www.hiv.lanl.gov/content/sequence/DRAW_CRF/recom_mapper.html

^6^ https://hivdb.stanford.edu/hivdb/bysequences/
